# Supplementary material for: Analytic Choices Shape Genomic Risk Estimates from Electronic Health Records: Coronary Heart Disease in eMERGE IV
Source: medRxiv. 2026 Apr 30:2026.04.28.26352002. Preprint. [Version 1] doi: 10.64898/2026.04.28.26352002 (PMC13142575; doi:10.64898/2026.04.28.26352002)
Supplement: 1 [file NIHPP2026.04.28.26352002V1-supplement-1.pdf]

medRxiv preprint doi: https://doi.org/10.64898/2026.04.28.26352002; this version posted April 30, 2026. The copyright holder for this preprint (which was not certified by peer review) is the author/funder, who has granted medRxiv a license to display the preprint in perpetuity.
It is made available under a CC-BY 4.0 International license .

SUPPLEMENTAL MATERIAL

Supplemental Table 1. Billing codes used in the phenotyping algorithm

See attachment

Supplemental Table 2. Basic characteristics of participants in the study cohort

	**Overall cohort (N = 11699)**
**Age at enrollment, yrs (mean± SD)**	51.21 ±14.92
**Age groups in years, n (%)**	
18-39	3065 (26.2)
40-49	2050 (17.5)
50-59	2355 (20.1)
60-69	2886 (24.7)
>70	1343 (11.5)
**Sex at assigned birth, n (%)**	
Female	7889 (67.4)
Male	3810 (32.6)
**Race and ethnicity, n (%)**	
Asian	967 (8.3)
Black, African American or African	1843 (15.8)
Hispanic, Latino or Spanish	1960 (16.8)
White	5999 (51.3)
Other/ Admixed/ Prefer not to answer	930 (8.0)
**Site, n (%)**	
Vanderbilt	680 (5.8)
UAB	2466 (21.1)
Columbia	1178 (10.1)
Mayo Clinic	1063 (9.1)
MPHC (part of Mayo)	278 (2.4)
UW	1942 (16.6)
Northwestern	1572 (13.4)
MGB	1025 (8.8)
Mount Sinai	1401 (12.0)
Cincinnati	42 (0.4)
CHOP	52 (0.4)
**High risk PRS, n (%)**	515 (4.4)
**Monogenic risk, n (%)**	83 (0.7)
APOB	14 (0.1)
LDLR	65 (0.6)
PCSK9	4 (0.0)

medRxiv preprint doi: https://doi.org/10.64898/2026.04.28.26352002; this version posted April 30, 2026. The copyright holder for this preprint (which was not certified by peer review) is the author/funder, who has granted medRxiv a license to display the preprint in perpetuity.
It is made available under a CC-BY 4.0 International license .

Supplemental Table 3. Chart review notes and conclusions for 15 participants with discordant CHD

**Self-report CHD**	**CHD by EHR algo**	**Relevant medical history from chart review**	**Chart review conclusion**
Self-reported CHD but not identified by phenotyping algorithm (10/36)			
Current CHD, stenting of heart arteries	None	Had MI dx 25 years prior to enrollment; stent placement (PCI) 12 years prior to enrollment.	Definitive CHD
Current CHD, stable angina	None	Receiving cardio care elsewhere because of chest pain, likely no CHD	**Likely no CHD**
Current CHD	None	Dx Long QT syndrome; had allergic reaction that mimic heart attack	**Likely no CHD**
Current and past CHD, heart attack, stenting	None	Had CAD with stent placement	Definitive CHD
Current CHD	None	Notes of arrythmia and ischemia; suspected of CAD because of calcification, but no evidence of MI, PCI, or CABG	**Likely no CHD**
Current CHD, heart attack, unstable angina	None	Currently followed by cardiology because of arrythmia, but no evidence of MI, PCI, or CABG	**Likely no CHD**
Current CHD	None	Only family history of heart disease noted. Limited chart information.	**Likely no CHD**
Current CHD	None	Has congenital coronary heart disease, but not atherosclerotic.	**Other CHD**
Past CHD	None	Has HHT, but cardiology visit noted “no coronary issues”	**Likely no CHD**
Current CHD	None	Palpitations, referred to cardiology, but no evidence of CAD	**Likely no CHD**
classified as CHD positive by the algorithm but self-reported no CHD			
None	MI	Based on a recent cardiology note, MI risks were discussed but cardiac catheterization indicated “no significant CAD”.	Likely no CHD
None	MI, PCI, CABG	Have CABG documented	**Definitive CHD**
None	MI, PCI	Have MI documented	**Definitive CHD**
None	PCI	Several MI documented; PCI documented	**Definitive CHD**
None	PCI	Vascular EDS, hepatic artery stent, iliac	Likely no CHD

medRxiv preprint doi: https://doi.org/10.64898/2026.04.28.26352002; this version posted April 30, 2026. The copyright holder for this preprint (which was not certified by peer review) is the author/funder, who has granted medRxiv a license to display the preprint in perpetuity.
It is made available under a CC-BY 4.0 International license .

		artery stent, notes indicated CAD prevention but no evidence of MI, PCI, or CABG	

Dx: Diagnosis, MI: Myocardial infarction, PCI

Supplemental Table 4. Cumulative incidence estimates [95% CI], under three alternative left truncation scenarios

	Left truncation scenarios		
Age (years)	**First billing code as entry**	**Start of most recent consecutive period as entry**	**No left truncation**
40	0.008 [0.005, 0.015]	0.015 [0.006, 0.037]	0.001 [0.001, 0.002]
45	0.016 [0.011, 0.024]	0.027 [0.016, 0.046]	0.004 [0.003, 0.006]
50	0.025 [0.019, 0.032]	0.039 [0.027, 0.057]	0.008 [0.006, 0.010]
55	0.043 [0.035, 0.051]	0.063 [0.049, 0.080]	0.018 [0.015, 0.021]
60	0.072 [0.063, 0.082]	0.099 [0.083, 0.117]	0.038 [0.033, 0.043]
65	0.110 [0.099, 0.122]	0.143 [0.127, 0.162]	0.070 [0.063, 0.078]
70	0.141 [0.129, 0.155]	0.179 [0.161, 0.198]	0.101 [0.091, 0.111]
75	0.188 [0.168, 0.209]	0.225 [0.202, 0.249]	0.149 [0.131, 0.169]
